# Cation Tuning toward the Inference of the Gelation Behavior of Supramolecular Gels

**DOI:** 10.1038/srep25390

**Published:** 2016-05-03

**Authors:** Peng Xue, Huiqiong Wu, Xiaojuan Wang, Ting He, Rujuan Shen, Fan Yue, Jide Wang, Yi Zhang

**Affiliations:** 1 Key Laboratory of Oil and Gas Fine Chemicals, Ministry of Education & Xinjiang Uygur Autonomous Region, Xinjiang University, Urumqi, Xinjiang 830046, China; 2 College of Chemistry and Chemical Engineering, Central South University, Changsha 410083, Hunan, China; 3 State Key Laboratory of Powder Metallurgy, Central South University, Changsha 410083, Hunan, China

## Abstract

We serendipitously discovered that the tripeptide Asp–Phe–Phe trifluoroacetic acid salt (hereafter abbreviated as β-AspFF) formed a reversible thermotropic gel in chloroform solution (at temperatures higher than the boiling point of chloroform), and a stable gel in toluene solution (at equal to or lower than the room temperature). Experimental results indicate that doping metal ions into β-AspFF toluene gels can trigger morphological variations in the gel skeleton, thereby increasing gel volume and inducing the collapse of organogels. Investigation on the cation-tuned gelation behavior of β-AspFF can be used to elucidate heating-induced gel collapse (of normal gel) or reverse thermotropic gelation as well as select carbamide and acetamide as activators of β-AspFF gels in chloroform solution at room temperature.

Advances in the use of supramolecular gelators for fabrication of polymer-like physical gels have inspired scholars to develop functional soft materials^1^,^2^,^3^,^4^,^5^. Well-designed low-molecular-weight gelators (LMWGs, <500 amu) contain necessary covalently linked moieties, which promote the growth of noncovalently associated building blocks through self-assembly^6^,^7^,^8^,^9^. Although LMWGs have been the research focus in supramolecular science, prediction of candidate gelator compounds remains unsuccessful because of the complex noncovalent interactions among individual gelators^10^,^11^.

The heat-set, jelly-like networks of LMWGs have attracted intensive attention as novel thermoresponsive materials. The reversible self-assembly of the first thermotropic LMWG is an enthalpically driven process, in contrast to the entropy-driven heat-set gelation of polymers and proteins^12^. Insufficient information on the structure–activity relationship of known thermotropic gels limits the development of new gelators.

“Molecular tunability” is universally used to fabricate objective three-dimensional (3D) structures through simple self-assembly because of the “weak force” maintained nature of supramolecular gels^13^,^14^,^15^,^16^. Cations or anions are used as a chemical stimulus to effectively elucidate the self-assembly pathway during gelation. Incorporating metals in supramolecular gels (metallogels) has also been extensively studied^17^,^18^. Furthermore, the binding effect of cations on supramolecular gels has begun to attract widespread attention^19^,^20^,^21^,^22^.

In this paper, we introduce a facile example of using cations as a chemical stimulus to investigate the structure–activity relationship of a thermotropic gel. The gelator β-AspFF is a tripeptide that contains diphenylalanine (FF) as the assembly core and aspartic acid moiety as the metal binding unit (Figure S1).

## Results

### Characterization of β-AspFF organogel

**Figure 1:**
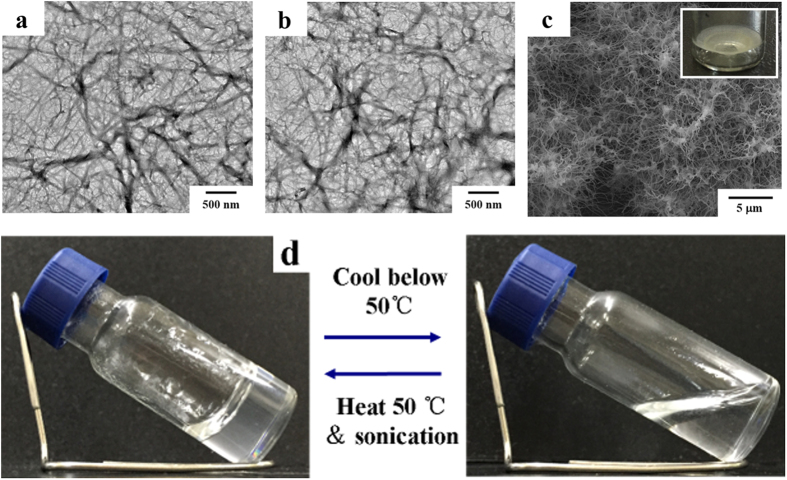
TEM images of cryo-dried scaffolds of β-AspFF nanofibrils at a concentration of 2 mg·mL^−1^ in different organogels (**a**) toluene organogel and (**b**) chloroform organogel); (**c**) SEM images of toluene gel after solvent evaporation; insert picture is a digital photo; and d: thermo-reversible gelation of β-AspFF gel at a concentration of 5 mg·mL^−1^ in chloroform.

The gelator formed gels in toluene and chloroform solutions. Interestingly, gels formed in the chloroform and toluene solutions are thermotropic and normal gels, respectively. The cryo-dried scaffolds of these gels exhibit well-organized 3D networks of nanofibrils (Fig. 1, detailed experiment in SI). Scanning electron microscopy (SEM) results showed that the β-AspFF toluene gel consists of fibrous networks with entangled long fibrils, with diameters ranging from 50 nm to 100 nm (Figure S2). This finding was confirmed by transmission electron microscopy (TEM) observations, in which fibrils possess an average diameter of less than 100 nm (Fig. 1a). Natural dried organogel in toluene generated umbrella-like (upside-down) polyporous residues at the bottom of the vessel; this gel still presents a 3D fibril network (Fig. 1c).

Cryo-dried gel formed in the chloroform solution exhibits morphological structure, similar to that of gels formed in the toluene solution, but manifests reverse thermodynamic behavior. Considering its limited information and distinct gelation pathway, thermotropic gel formation using LMWG must be investigated. However, imaging tools, such as SEM, TEM, and atomic force microscopy (AFM), cannot provide *in situ* structural information about gelation. This limitation is particularly irremediable for studying the structure–activity relationship of thermotropic gels.

### Nano-assemblies of β-AspFF in different solvents

**Figure 2:**
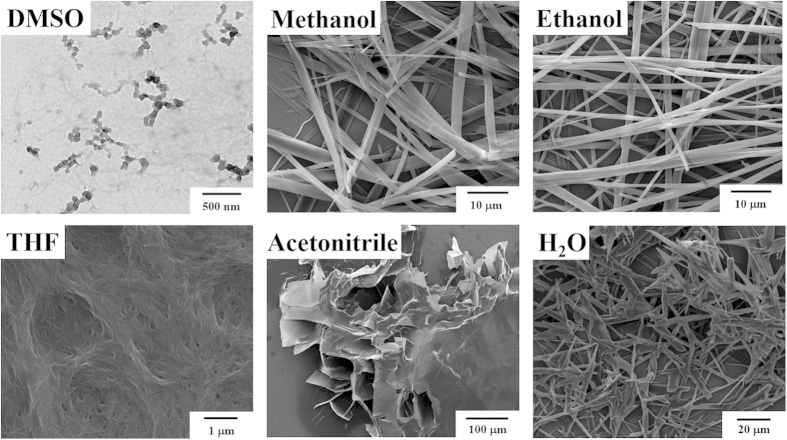
Morphologies of cryo-dried scaffolds of β-AspFF aggregates assembled in different solvents (at a concentration of 10 mg·mL^−1^ in DMSO, methanol, ethanol and THF; at a concentration of 2 mg·mL^−1^ in acetonitrile and deionized H_2_O).

The nano-assemblies of β-AspFF in solvents were first characterized (Fig. 2). At room temperature, β-AspFF self-assembled into nanoribbon structures in methanol, nanospheres in DMSO, nanofibers in ethanol, nanosheet assemblies in acetonitrile, and microtape structures in deionized H_2_O. The SEM results indicated that β-AspFF nanoaggregates were formed in water, and THF exhibits highly similar structures to those of nanofibril interactions in the toluene gel. Hence, formation of fibrillar structures may be a necessary condition but insufficient for gel formation.

The physical and chemical properties of solvents play important roles in gelation. In this study, we summarized several main solvent parameters, such as polarity parameter, hydrogen bond donor (HBD) ability, hydrogen bond acceptor (HBA) ability, and surface tension^23^.

Table 1 presents that β-AspFF prefers to be gelled in solvents with low HBD and HBA values^24^. This finding confirmed that β-AspFF tends to self-assemble to well-organized aggregates through inter-molecular π–π and hydrogen bond interactions. By contrast, the intermolecular hydrogen bond interaction between gelator and solvent molecules weakened in solvents with low HBD and HBA abilities. Thus, the inter-molecular hydrogen bond interaction in β-AspFF organogels is not the main driving force to trap and immobilize large volumes of solvent molecules.

The nature of the initial solvent (stock solution) also plays a key role in the gelation pathway^25^,^26^. The diphenylalanine peptide formed nanotubes / nanowires in hexafluoroisopropanol (HFIP)–H_2_O but generated intertwined nanofibrils in pure H_2_O. The experimental results suggest that the interaction between the initial solvent and the main solvent primarily affects β-AspFF gel. For instance, β-AspFF forms intertwined nanofibrous networks when first dissolved in HFIP. Followed by dilution with toluene or chloroform to a final concentration of 3 mg·mL^−1^; these networks could bind solvent molecules to the gel. We replaced HFIP with DMSO as the initial stock solvent and failed to obtain organogels in toluene and chloroform solutions. Only dispersion nanosticks were observed in the systems, as shown in Figure S3 and Table S1.

The fabricated organogel is sensitive to organic acids and bases. Adding one drop (10 μL) of triethylamine (TEA) into a preformed β-AspFF gel can trigger sol transition. The TEM observations suggested that nanofibrils in the β-AspFF gel are partly converted into nanospheres after TEA addition (Figure S4a). One drop of trifluoroacetic acid (TFA) can transform the gel into a clarified solution after several minutes. The TEM images revealed that the clarified solution contains irregularly shaped nanoparticles (Figure S4b). Significant pH changes can trigger 3D morphological rearrangements of the gelator, leading to failure to maintain network structure and finally inducing gel collapse. Hence, the nanofibril structure is necessary for efficient grasping of solvent molecules by the gelator to form a gel.

### Doping of metal ions into the gel system

**Figure 3:**
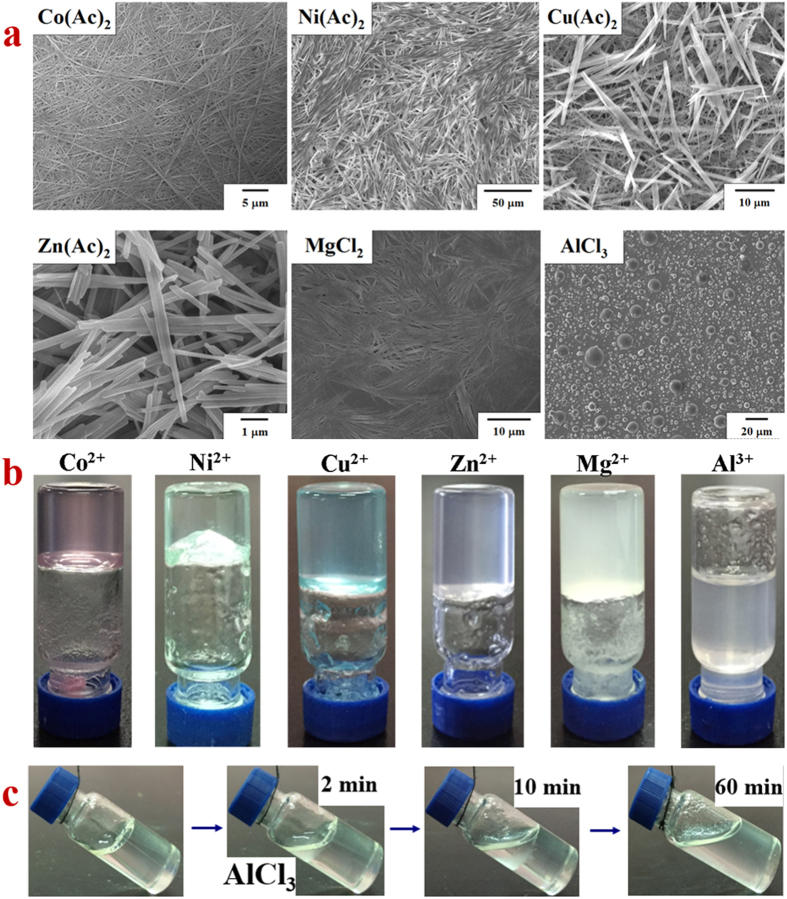
Images of β-AspFF toluene gel doped with 10 mg·mL^−1^ metal ions (**a**) SEM images and (**b**) Digital images); (**c**) Collapse procedure digital photos of β-AspFF toluene gel doped with 10 mg·mL^−1^ AlCl_3_.

To investigate the relationship between the structural features and gelation ability of β-AspFF, we added metal ions into the gel system under acidic conditions (to avoid coordination effect). Our experimental results confirmed that β-AspFF formed gels in acidic toluene solution in the presence of several metal ions, such as Co^2+^, Ni^2+^, Cu^2+^, Zn^2+^, and Mg^2+^ (Fig. 3).

The SEM images confirmed that the β-AspFF gelator doped with Co^2+^ assembled into nanofibers and minimally differed from pure β-AspFF gel. Ni(Ac)_2_-doped organogel are mainly composed of well-organized fibrils, and the final volume of the gels slightly increased compared with that of Co(Ac)_2_–doped organogel.

Gels doped with Cu^2+^, Zn^2+^, and Mg^2+^ possess larger volumes but poorer appearance compared with Co^2+^ –doped gel. The SEM observations suggested that gels doped with Cu^2+^ and Zn^2+^ are supported by 3D networks of shorter but more rigid nano-bars, whereas Mg^2+^ -doped gel is composed of bundled nanosticks. Evidently, the 3D arrangements of gels doped with Cu^2+^, Zn^2+^, and Mg^2+^ are considerably less compact than that of Co^2+^ -doped gel. The cryo-dried skeleton of gels doped with Cu^2+^ and Zn^2+^ share highly similar structures to those of pure β-AspFF aggregates in methanol and ethanol solutions.

In contrast to divalent metal ions, Al^3+^ -doped β-AspFF toluene system cannot form a gel; moreover, addition of a small amount of AlCl_3_ into the well-maintained pure β-AspFF toluene gel could lead to gel collapse. The SEM images of the cryo-dried sample suggested that Al^3+^ -doped β-AspFF formed micro-sized spherical particles. After sonication-induced gelation, permeate dispersion M(Ac)_2_–doped organogel exhibited a clearer and cleaner appearance than MCl_2_–doped system. Metal ions (M = Co^2+^, Ni^2+^, Cu^2+^, Zn^2+^) feature similar ionic radius. [CH_3_COO]^−^ possesses the smallest ionic radius among the ions that were distributed in the gel channel, and Co(Ac)_2_, Ni(Ac)_2_, Cu(Ac)_2_, and Zn(Ac)_2_ present similar transparency. Meanwhile, Cl^−^ shows large ionic radius, which could block the pore and appear turbid. However, the size of Al^3+^ is smaller than those of the other ions. We assumed that the β-AspFF gel skeleton could not bound to Al^3+^; thus, free ions wander to break the gel skeleton into nanospheres, resulting in gel collapse (the ionic radii of Co^2+^, Ni^2+^, Cu^2+^, Zn^2+^, Mg^2+^, Al^3+^, and Cl^−^ are 75, 70, 73, 75, 72, 53, and 181 pm, respectively).

### Doping of urea into the β-AspFF chloroform solution

**Figure 4:**
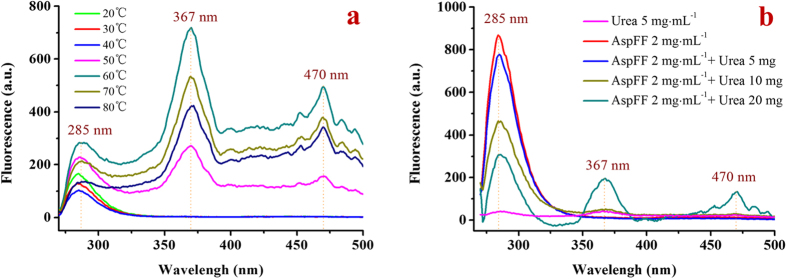
Fluorescence emission (*λ* excitation = 260 nm) of β-AspFF chloroform sol. (**a**) different temperature at 2 mg·mL^−1^ β-AspFF and (**b**) added urea at 293 K).

We selected urea to increase the enthalpy of β-AspFF chloroform system (thermotropic gel)^27^,^28^. Our experimental tests revealed that urea is an effective reagent used to decrease the gelation transition temperature of β-AspFF in chloroform. Fluorescence spectrum was also used to trace the *in situ* structural changes of β-AspFF during heat- and urea-induced gelation. Figure S5 shows that, at room temperature, fluorescence signal increased with increasing β-AspFF concentration within low ranges (<1 mg·mL^−1^). The strongest fluorescence signal appeared at 1 mg·mL^−1^, and further increase in the concentration of β-AspFF caused the aggregation-induced fluorescent quenching phenomenon. Below the gelation temperature (<323 K), fluorescence signal minimally changed with increasing temperature (Fig. 4a). An abrupt fluorescence peak appeared at 367 nm when heated at 323 K; continuous heating of the system (323 K to 333 K) led to enhanced fluorescence strength. The gel transferred to a clear sol at 353 K, whereas the corresponding fluorescence peaks at 367 and 470 nm significantly decreased. This finding indicated that peaks at 367 and 470 nm are due to gelation-induced fluorescence enhancement.

We used a sample with β-AspFF to determine changes in fluorescence signals in urea-induced β-AspFF gelation. Characteristic peaks at 367 and 470 nm emerged with increasing urea concentration. A remarkable parallel result during heating above 323 K confirmed that addition of urea to the β-AspFF chloroform system may induce structural transformations similar to those of heating at 323 K.

The cation-induced gelation behavior is highly similar to our observations when heating chloroform gel. The key advantage of using cation-induced gelation to mimic heat-induced gel collapse or reverse heat-set gelation is that well-controlled intermediate structures can be obtained in heating-related gelation by carefully tuning the concentration of cations.

Theoretically, increase in temperature of a system will accelerate molecular motion for all species, referring to a process of entropy increase^28^. However, gel formation is a phenomenon that requires entropy reduction. As such, increased temperature could induce gel collapse rather, than sol-to-gel transition, unless the energy reduction of structural changes caused by heating can compensate the entropy production effect.

## Discussion

The lack of well-developed experimental techniques to monitor the intermediate structure of gelators in a heating process considerably limits studies on the structure–activity relationship of thermotropic LMWG gelator. To date, most known thermotropic gels are polymer gels, and LMWG gel are stable at temperatures equal to or lower than the room temperatures. In this study, we introduced a simple strategy of using metal ions to progressively tune the morphological structure of the nano-assemblies of peptidic LMWG and consequently adjust the gelation behavior of the thermotropic LMWG gelator. Our experimental results confirmed the importance of special morphological nanostructures in gel stability. This unique gelation property renders β-AspFF as a potent template for studies on the distinctive gelation pathway of thermotropic gelators, particularly LMWG gelators.

## Methods

### Procedure for gel preparation

The β-AspFF tripeptide was first dissolved in 1,1,1,3,3,3-hexafluoro-2-propanol (HFIP). Subsequently, 50 μL of 0.1 M β-AspFF / HFIP solution was diluted to a final concentration of 5 mM in toluene or chloroform solution. Sonication for 5 minutes could obtain gels, whereas chloroform gels required longer time and were found at more than 323 K.

### Nano-assemblies in different solvents

β-AspFF 100 mg·mL^−1^ HFIP stock-solutions were prepared and diluted to 10 mg·mL^−1^ by adding dissolvent or 2 mg·mL^−1^ solvent with poor solubility. The samples were incubated for an hour and then dropped to silicon wafer. After drying, electron microscopy analyses were performed.

### Doping of metal ions into the β-AspFF toluene gel system

Briefly, 10 mg·mL^−1^ metal ions were dissolved in 50 μL of HFIP and then doped into 5 mM β-AspFF toluene gel. The dispersion was permeated for several hours to obtain various metal ion gels, except Al^3+^.

### Doping of urea into theβ-AspFF chloroform solution

A certain concentration of the urea solution was dispersed in chloroform, and the final volume was adjusted to 950 μL. The urea solution was then added to 50 μL of 40 mg·mL^−1^ β-AspFF/HFIP solution.

## Additional Information

**How to cite this article**: Xue, P. *et al.* Cation Tuning toward the Inference of the Gelation Behavior of Supramolecular Gels. *Sci. Rep.* **6**, 25390; doi: 10.1038/srep25390 (2016).

## Supplementary Material

Supplementary Information

## Figures and Tables

**Table 1:** Property parameters of pure organic solvents.

Solvent	Polarity	HBD	HBA	Surface	Assemblies
	index	ability	ability	tension	morphology
Toluene	2.40	0.00	0.11	28.53	Nanofiber[a]
Chloroform	4.10	0.20	0.10	27.16	Nanofiber[a]
THF	4.2	20.0	8.0	26.4	Nanofiber
EA	4.3	17.1	9.3	26.29	Nanofiber
Ethanol	4.3	0.86	0.75	22.27	Nanofiber
Acetonitrile	6.2	0.19	0.4	19.1	Nanosheet
Methanol	6.6	0.98	0.66	22.55	Nanofiber
DMSO	7.99	29.8	19.3	43.6	Nanosphere
Water	10.20	1.17	0.47	72.80	Microtapes

^[a]^The phase behavior is organogels.
