# Supplementary material for: Cation Tuning toward the Inference of the Gelation Behavior of Supramolecular Gels
Source: Sci Rep. 2016 May 3;6:25390. doi: 10.1038/srep25390 (PMC4853806; doi:10.1038/srep25390)

**Supporting Information**

Cation Tuning toward the Inference of the Gelation Behavior of Supramolecular Gels

Peng Xue^1^, Huiqiong Wu^2^, Xiaojuan Wang^2^, Ting He^2^, Rujuan Shen^3^, Fan Yue^1^, Jide Wang^1,*^, and Yi Zhang^2,*^

^1^Key Laboratory of Oil and Gas Fine Chemicals, Ministry of Education & Xinjiang Uygur Autonomous Region, Xinjiang University, Urumqi, Xinjiang 830046, China

^2^College of Chemistry and Chemical Engineering, Central South University, Changsha 410083, Hunan, China

^3^State Key Laboratory of Powder Metallurgy, Central South University, Changsha 410083, Hunan, China.

E-mail: yzhangcsu@csu.edu.cn; awangjd@sina.cn

**Figures and tables**

**Figure S1** Schematic representation of the molecular structure of the studied β-AspFF.


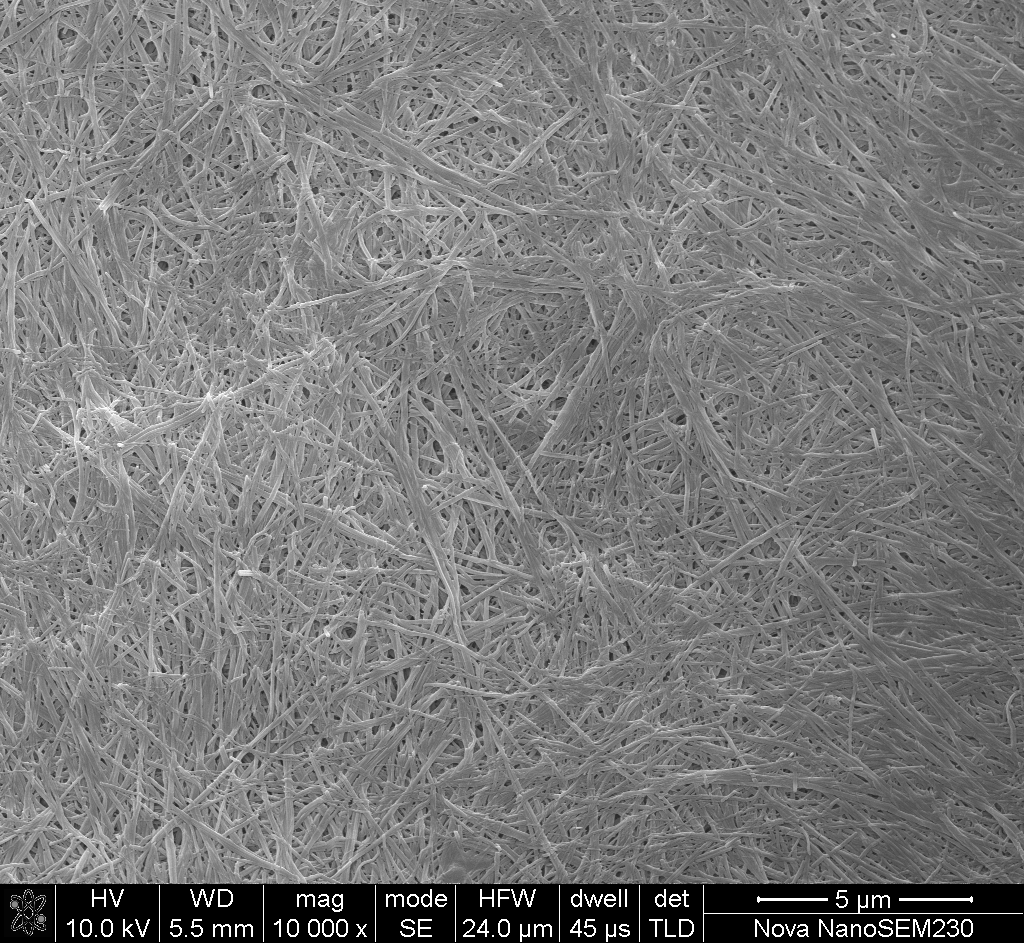


**Figure S2** SEM image of cryo-dried scaffold of β-AspFF nanofibrils at the concentration of 10 mg·mL^-1^ in toluene


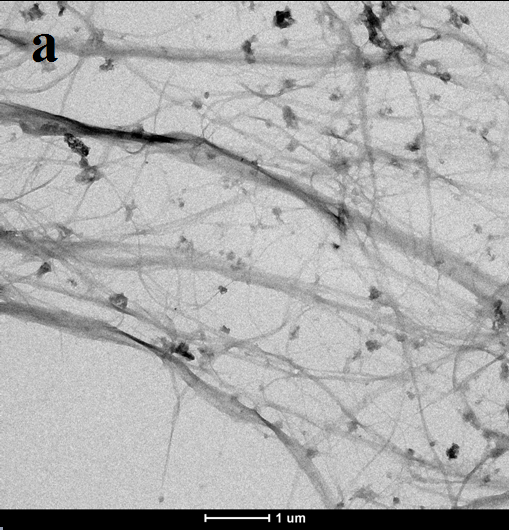


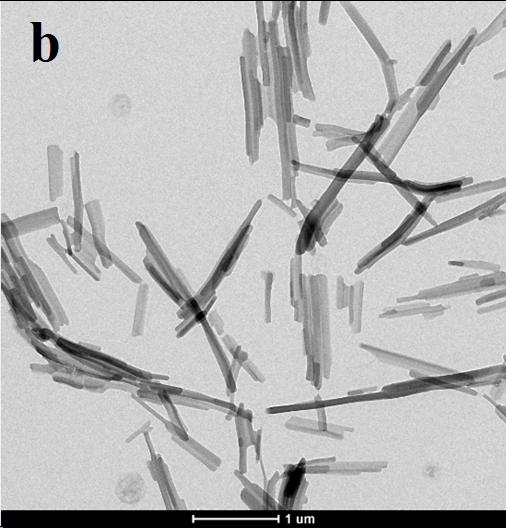


**Figure S3** TEM images of β-AspFF gel at the concentration of 2 mg·mL^-1^ in (a: HFIP-H_2_O and b: DMSO-toluene)


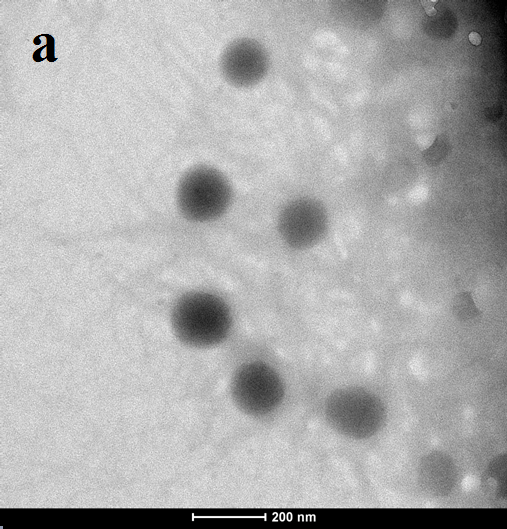

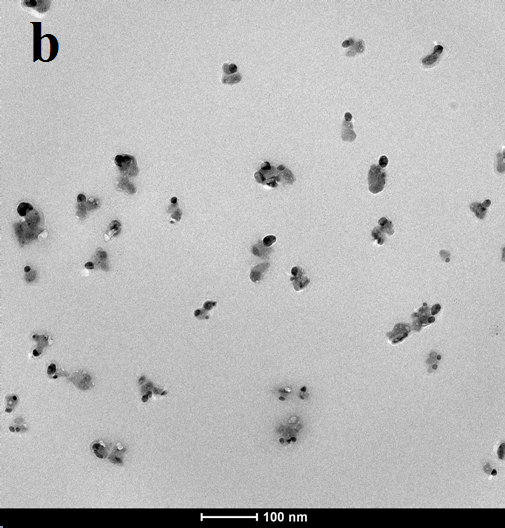


**Figure S4** TEM images of β-AspFF systems at the concentration of 6 mg·mL^-1^ at different pH value (a: TEA and b: TFA)


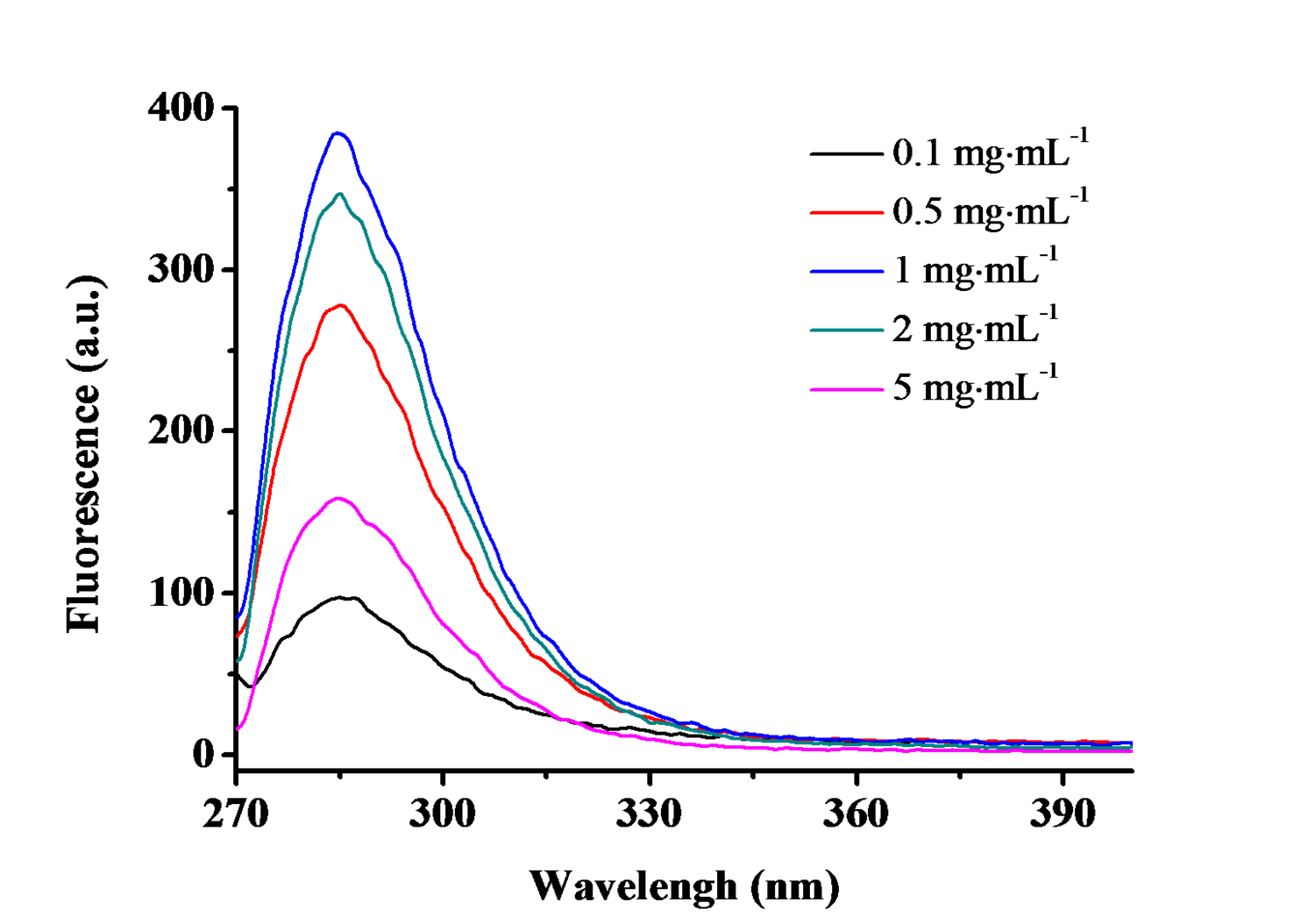


**Figure S5** Florescence emission (*λ*excitation 260 nm) of different concentrations of β-AspFF chloroform sol.

**Table S1** Two kinds of protocol to prepare gel.


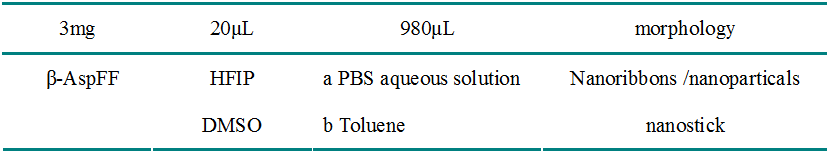

Supplement: Supplementary Information [file srep25390-s1.doc]
